# Deep learning with mixup augmentation for improved pore detection during additive manufacturing

**DOI:** 10.1038/s41598-024-63288-1

**Published:** 2024-06-11

**Authors:** Bulbul Ahmmed, Elisabeth G. Rau, Maruti K. Mudunuru, Satish Karra, Joshua R. Tempelman, Adam J. Wachtor, Jean-Baptiste Forien, Gabe M. Guss, Nicholas P. Calta, Phillip J. DePond, Manyalibo J. Matthews

**Affiliations:** 1 Earth and Environmental Sciences Division, Los Alamos National Laboratory, Los Alamos, NM 87545 USA; 2 Matador Resources Company, Dallas, TX 75240 USA; 3 Subsurface Science Group, Pacific Northwest National Laboratory, Richland, WA 99352 USA; 4 Environmental Molecular Sciences Laboratory, Pacific Northwest National Laboratory, Richland, WA 99352 USA; 5 Engineering Institute, Los Alamos National Laboratory, Los Alamos, NM 87545 USA; 6 Mechanical Science and Engineering, University of Illinois at Urbana-Champaign, Urbana, IL 61801 USA; 7 Physical and Life Sciences Directorate, Lawrence Livermore National Laboratory, Livermore, CA 94550 USA; 8 Engineering Directorate, Lawrence Livermore National Laboratory, Livermore, CA 94550 USA

**Keywords:** Additive manufacturing, Pore formation, Deep learning, Convolutional neural networks, High-throughput data, Imbalanced learning, Imaging techniques, Applied mathematics, Engineering

## Abstract

In additive manufacturing (AM), process defects such as keyhole pores are difficult to anticipate, affecting the quality and integrity of the AM-produced materials. Hence, considerable efforts have aimed to predict these process defects by training machine learning (ML) models using passive measurements such as acoustic emissions. This work considered a dataset in which keyhole pores of a laser powder bed fusion (LPBF) experiment were identified using X-ray radiography and then registered both in space and time to acoustic measurements recorded during the LPBF experiment. Due to AM’s intrinsic process controls, where a pore-forming event is relatively rare, the acoustic datasets collected during monitoring include more non-pores than pores. In other words, the dataset for ML model development is imbalanced. Moreover, this imbalanced and sparse data phenomenon remains ubiquitous across many AM monitoring schemes since training data is nontrivial to collect. Hence, we propose a machine learning approach to improve this dataset imbalance and enhance the prediction accuracy of pore-labeled data. Specifically, we investigate how data augmentation helps predict pores and non-pores better. This imbalance is improved using recent advances in data augmentation called Mixup, a weak-supervised learning method. Convolutional neural networks (CNNs) are trained on original and augmented datasets, and an appreciable increase in performance is reported when testing on five different experimental trials. When ML models are trained on original and augmented datasets, they achieve an accuracy of 95% and 99% on test datasets, respectively. We also provide information on how dataset size affects model performance. Lastly, we investigate the optimal Mixup parameters for augmentation in the context of CNN performance.

## Introduction

Additive manufacturing (AM) is a process of creating objects by adding layers of material. AM has revolutionized manufacturing in the medical, aerospace, and automotive industries because of its wide range of applications, from large-scale production to one-of-a-kind objects doing all with low material consumption^1–4^. With the AM technology, one can design and develop custom designs and complex shapes that are difficult or impossible through conventional production means^5^.

**Figure 1:**
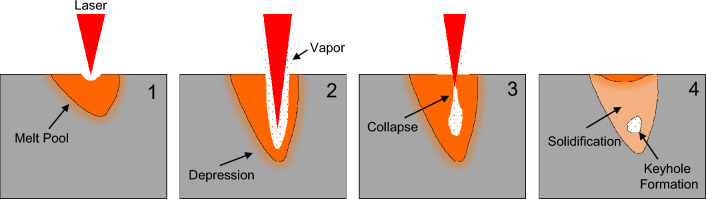
Schematic of the formation of a keyhole pore during the LPBF process. (1) Formation of the initial melt pool. (2) Formation of depression. (3) Collapse of the materials along the walls of the depression due to overheating. (4) Formation of a keyhole pore with entrapped vapor and the surrounding material undergoes rapid solidification.

Laser powder bed fusion (LPBF) is an AM technology that fuses powder material to form three-dimensional objects. One of the primary challenges in LPBF-enabled AM manufactured parts is the reproducibility of the design due to defect formation^5^. Some common defects associated with LPBF are surface roughness, dimensional accuracy, balling, cracking, and formation of unintentional pores^1,6–8^. Porosity defects are particularly critical because they have a significant impact on fatigue performance^9^. During the LPBF process, a depression, which can transition to a keyhole, forms due to metal vaporization (Fig. 1)^10^. Under certain conditions, this keyhole can become unstable, causing bubbles in the melt pool^11,12^. Rapid solidification of the melt pool traps these bubbles in the solid part, resulting in pores that remain in the final part, known as keyhole pores. This formation of pores can cause significant quality issues for the manufactured part, such as sub-par mechanical behavior. Methods such as X-ray tomography are available to determine the size and location of pores ex situ. However, this technique is complex and costly, and the intricate geometries of additive manufacturing only sometimes allow for straightforward imaging. Hence, pore defects must be determined, preferably *in situ*, to remediate the formation by modifying the LPBF process parameters.

Recently, machine learning (ML) techniques have been used to classify pores and non-pores from acoustic emissions^13–21^. Pandiyan et al., 2020^19^ performed blind clustering to demonstrate that pore-associated LPBF sintering conditions cluster in a spectra-based feature space. However, they could not localize these findings because no spatiotemporal registration exists within their work. Tempelman et al.^14^ trained and tested an ML model utilizing a support vector machine (SVM) on a spatiotemporal registered acoustic data of LPBF. The high-throughput acoustic data were collected using a microphone in the print chamber at 100kHz. They were able to classify keyhole pores from the waveforms with an accuracy of up to 97%. Another key finding of this research is that spectral featurization (e.g., frequency bands) was essential for predicting pore formation. Further studies^14^ investigated the role of power spectral densities (PSDs) on pore detection. Specifically, on this same acoustic dataset, Tempelman et al.^14^ used non-negative matrix factorization with customized *k*-means clustering (NMF*k*)^22–24^ to correlate PSDs with pore formation. An outcome of this analysis is that NMF*k* blindly (no defined outputs) confirms that the power spectra are the important spectral patterns to detect pore formation. This NMF*k* decomposition projects the high-throughput data onto a lower-dimensional space, allowing further processing and development of latent feature space or key signatures, successfully classifying the pore formation. The discovered pore formation signature was a broadband signal with dominant frequencies between 10 kHz and 40 kHz that agrees with the work by Pandiyan et al., 2020^19^. The lower-dimensional projected data was then used to build a series of unsupervised and supervised ML classifiers. Classification of our test datasets was performed based on a supervised ($\sim$95% accuracy) and unsupervised ($\sim$90% accuracy) training labeling scheme and using a suite of ML classifiers. More recently, computational studies performed byKhairallah et al. ^25^ have predicted the onset of melt-pool oscillations immediately preceding keyhole formation in a similar frequency range to those found important in the References^13,15,19^.

In a recent study, Ren et al.^20^ performed computational predictions of melt pool oscillations, which were corroborated with thermal measurements of the melt pool in a real-time X-ray radiography experiment to train a machine learning model. This resulted in near-perfect predictions of in situ pore detection for single-scan experiments, which delivered invaluable context to the dynamics of the melt pool as pore formation occurs and validated the frequency-driven approaches of previous acoustics studies. However, this study was limited to single-track scans in an X-ray beamline setting rather than commercial-level builds, although a compelling data registration scheme was presented for future works.

To this end, the focus of ex situ registered experiments with commercially relevant datasets remains a topic worthy of further study. In the context of passive acoustics-based monitoring for pore-registered commercial-level experiments^14,15^), accuracy scores are likely not to represent pores because the number of pores is fewer than non-pores due to the intrinsic control of the AM process. Because the likelihood of a pore-forming event is less than non-pore, the labeled acoustic data has more non-pore samples than pore samples, naturally leading to an imbalanced problem^26^. Moreover, datasets that register pores locations to AM process measurements are notoriously small in size and sparse in the number of recorded pores, as such data collection campaigns remain highly nontrivial endeavors^26^. For such a dataset, accuracy scores covering both pores and non-pores may mispredict new datasets. Additional data needed to balance an existing dataset can also be expensive or inaccessible to collect to avoid such pitfalls. Data augmentation, oversampling, and undersampling techniques are generally used to address imbalanced data^27–32^. Since one would ideally like to utilize as much of the data as possible, it is disadvantageous to use undersampling on smaller datasets. Because the datasets used in this study are small, we use oversampling to tackle the class imbalance. The effectiveness of oversampling data augmentation in enhancing the ML model’s training and prediction for AM processes has yet to be compared between the original and augmented datasets.

Data augmentation techniques have been increasingly applied in additive manufacturing research to enhance the performance and generalization of machine learning models. These methods aim to artificially increase the size and diversity of the training dataset by applying various transformations to the existing data. Some common data augmentation techniques used in additive manufacturing include geometric transformations, such as rotation, scaling, and flipping^33^, which help the ML model learn invariance to these transformations. Another approach is the use of generative adversarial networks (GANs) to synthesize new, realistic samples that mimic the distribution of the original data^34–37^. While these techniques have shown promising results, they may not effectively address the challenges of imbalanced datasets, where certain classes (e.g., defects) are significantly underrepresented. In contrast, the Mixup algorithm^38^ directly tackles the class imbalance problem by creating new samples through a weighted combination of input features and their corresponding labels. Mixup generates a more balanced and diverse dataset by interpolating between samples from different classes, enabling the ML model to learn more robust decision boundaries. This makes Mixup particularly well-suited for additive manufacturing applications, where the detection and classification of rare defects or anomalies are of critical importance.

The primary objectives of this study are: (1) apply ML on original and augmented acoustic datasets to predict pore formation, (2) provide performance metrics in terms of pore and non-pore formation events, and (3) provide a guideline on when to use original or augmented data for training ML models. We trained and tested a convolutional neural network (CNN) utilizing one-dimensional kernels and filter sizes on five different acoustic datasets collected during LPBF experiments to achieve this objective. We performed data augmentation using Mixup^38^, a weakly supervised labeling process on this collected experimental data. The performance of the trained CNN model is expressed in: (1) F1 scores for pore and non-pore formations and (2) accuracy (combined F1 scores for both pore and non-pore formations) along with the non-pore to pore ratio control in all five datasets. Furthermore, we describe how the dataset size impacts CNN model performance and then assess the effect of data augmentation on ML model performance/efficiency. Finally, we propose guidelines for when a user can use original or augmented data for in situ monitoring of the AM process.

## Methods

### Data acquisition and processing

**Figure 2:**
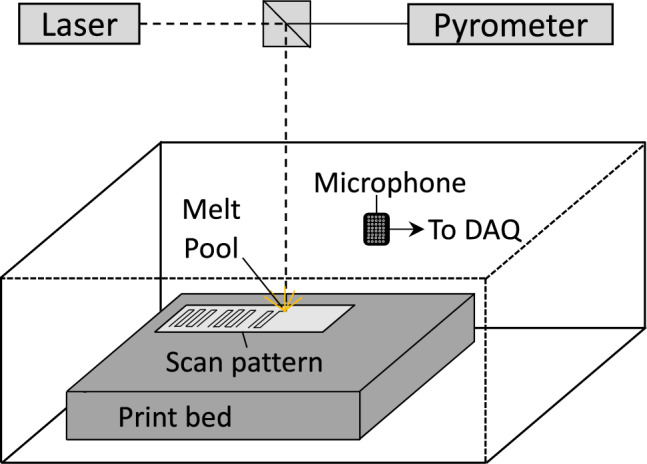
A schematic of an experiment to collect acoustic emission data during the LPBF process to assess pore formation.

**Figure 3:**
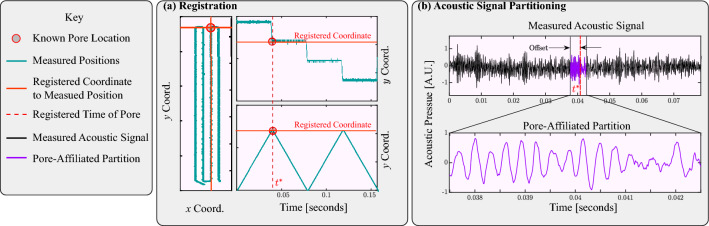
The spatial-temporal registration scheme involves two main steps. First, the pore location(s) identified in radiography images are registered to the corresponding laser-measured locations on an $x-y$ coordinate grid (**a**). Second, the time(s) at which the measured *x* and *y* coordinates align with a registered pore are denoted as $t^*$, representing the registration time(s) (**b**). The acoustic signal is then partitioned into a pore-affiliated segment based on the registered pore time (*t*) and a random offset. This segmentation allows for the isolation and analysis of the acoustic data associated with the registered pore(s).

The LPBF experiments were performed at Lawrence Livermore National Laboratory with variable laser powder and speed, which were presented in^13–15^. The system utilized a 1070 nm continuous wave 400 W Yb-fiber with a beam diameter of approximately 100 μm at the focal point. The material used in all studies was 316L stainless steel powder with 50 μm layer thickness placed on 316L plates. Single patches measuring between $2\times 5$ mm^2^ and $1\times 5$mm^2^ were melted with laser powers ranging between 50–375 W and scan speed between 100 and 400 mm/s at a 100 μm hatch spacing. The physical distance that the beam has moved is between 1 and 4 mm based on laser speeds between 100 and 400 μm/s. A microphone recorded acoustic measurements affixed to the build chamber positioned approximately 25 cm from the center of the build plate. Data was recorded at a sampling rate of 100 kHz with an AC-coupled low-pass filter at 6 dB via a Stanford Research System preamplifier. The distance from the microphone to the tracks was far greater than the size of the lasing tracks themselves, meaning that acoustic measurements were largely agnostic to the specific positions or direction of the laser. Acoustic emissions of the build process are recorded using an acoustic transducer synchronized to the x and y coordinates of the laser using a similar experimental design described in Reference^39^ and data registration scheme described in Reference^13^. The schematics presented in Figs. 2 and 3 illustrate the data acquisition and co-registration process. Data are collected at a sampling rate of 100 kHz with an AC-coupled low pass filter of 6 dB and a 10X gain via a Stanford Research Systems preamplifier. Post-build X-ray radiography was performed at beamline 8.3.2 of the Advanced Light Source at Lawrence Berkeley National Laboratory to identify the spatial locations of pores ex-situ. Then, the laser position synchronizes pores with the corresponding acoustic emission time histories. An important limitation of this experimental dataset is that it only looks at single-layer scans and cannot probe the lack of fusion pore formation events.

The acoustic data was divided into 10 ms windows and labeled as either pore or non-pore based on radiography images. The start of the acoustic waveform windows was offset so that the pores appeared randomly within the 10 ms window. An additional offset was included to compensate for the time-of-flight of the acoustic signal based on the speed of sound in Argon gas with an assumed distance of 25 cm separating the melt pool from the acoustic sensor. This window selection ensures that the pores sometimes occur within the same position in their corresponding acoustic window. This acoustic window partitioning replicates an in situ monitoring scheme where data is likely to be randomly partitioned into segments without knowing the location of a possible pore before ML analysis. The remaining acoustic emissions are labeled as non-pores.

Acoustic data alignment (or co-registration)^40^ with the pores has already been performed before the data is provided to ML model training. To account for the time-of-flight delay, we apply an offset when collecting time series partitions affiliated with pores based on the speed of sound in Argon and an approximate distance of 25 cm between the acoustic source and the microphone (roughly a 0.77 ms delay). Table 1 reports the acoustic microphone calibration data. Additionally, please refer to Sec-2 and references within Tempelman et al.^14^, which provides more information on the acoustic emission data collection process from the LBPF experiment.

**Table 1:** Acoustic microphone calibration data.

Maximum sound level (dB SPL)	Sound pressure (Pa)	Output signal	Noise floor measured (V)	Noise floor mic (mV)	Dynamic range (SNR)	Dynamic range (dB)
120	20	159.62	0.1	2	79.81	83.04

### Data description

We used acoustic datasets from five different experimental trials to train and test ML models, each corresponding to a different substrate used in the LPBF machine. In addition, we also used a combination of all these five datasets for training and testing, which we refer to as a combined dataset. Table 2 summarizes the details of the training and testing data split and the pore/non-pore labels. Each of these samples within the 10 ms window had a time series of 1000 points. The trained ML models are then used to classify the test data samples as either pore or non-pore. All five experimental acoustic datasets have a different number of pores and non-pores and exhibit class imbalance. The non-pore-to-pore ratio of acoustic sets 1, 2, and 4 is greater than 3, while the ratio is less than 2 for acoustic sets 3 and 5.

Note that each set (i.e., 1, 2, $\ldots$, 5) refers to an individual LPBF experiment. During the experiment, acoustic emissions are collected for each set. As the collected data is imbalanced, stratification is performed to split this data into two sets: training (imbalanced) and testing (balanced). That is, stratified sampling offers a significant advantage in this context. Since the population density of pores (air pockets within the material) varies significantly across different regions within each experiment, stratified sampling ensures the ML model receives a balanced representation of these variations. This, in turn, helps the CNNs trained on this data achieve more consistent accuracy across different regions of the LPBF experiments, as shown in Fig. 7, Tables 3 and 4. Furthermore, combining the acoustic data from all five experiments into a single training set allows us to compare the predictive performance of the CNNs across different sets. This comparative analysis provides valuable insights into the models’ generalizability in the LPBF process. Undersampling techniques can be employed alongside stratified sampling to address the class imbalance within the training set. Undersampling reduces the representation of the majority class to match the size of the minority class. The testing set size is carefully chosen to ensure it contains enough samples for a statistically robust evaluation of CNN’s generalizability under limited and imbalanced training data. By incorporating such a data preprocessing step, we can ensure the ML models are trained on a balanced and representative dataset, ultimately leading to more robust and generalizable CNN predictions for real-world LPBF applications.

**Table 2:** A summary of training and testing datasets for developing ML models.

Set	Training data				Testing data			
	Samples	Pores	Non-pore	Ratio	Samples	Pores	Non-pores	Ratio
1	370	73	297	4.1	1288	644	644	1.0
2	222	42	180	4.3	1376	688	688	1.0
3	703	249	454	1.8	1088	544	544	1.0
4	415	98	317	3.2	1261	630	631	1.0
5	806	360	446	1.2	975	462	513	1.1
Combined	2516	822	1694	2.06	6038	3019	3019	1.0

The five sets refer to the data acquired from five different LPBF experiments. The details of the combined dataset are also shown. Training data are the samples used to train the model, and the testing data are used as blind data to test overall ML model performance. Non-pore to pore ratio refers to the number of data points mixed to make a balanced dataset.

**Table 3:** ML model results comparing original data to one $\lambda$ for data augmentation.

Acoustic set		Original			Augmented		
#	T. ratio	F1 pore	F1 non-pore	Accuracy	F1 pore	F1 non-pore	Accuracy
1	4.1	0.92	0.93	0.93	0.94	0.95	0.94
2	4.3	0.93	0.93	0.93	0.94	0.94	0.94
3	1.8	0.94	0.93	0.94	0.95	0.95	0.95
4	3.2	0.89	0.91	0.90	0.90	0.92	0.91
5	1.2	0.94	0.94	0.94	0.92	0.93	0.93
Combined	2.06	0.95	0.95	0.95	0.99	0.99	0.99

Here, T. ratio refers to non-pore to pore ratio in each acoustic dataset.

**Table 4:** Comparison of true positive (TP), true negative (TN), false positive (FP), and false negative (FN) classifications of original datasets and augmented datasets (with one $\lambda$ value) for each respective acoustic set.

Set	Original				Augmented			
	TP	TN	FP	FN	TP	TN	FP	FN
1	562	630	14	82	584	632	12	60
2	618	657	31	70	645	654	34	43
3	500	523	21	44	507	526	18	37
4	515	621	10	115	522	627	4	108
5	430	488	25	32	406	499	14	56
Combined	2853	2875	166	144	2990	3014	29	5

### Data augmentation

Mixup data augmentation is a technique that enhances ML model performance by addressing class imbalance problems and promoting better generalization^32,38,41,42^. In the Mixup approach, given a training dataset with input and output, augmented data is created by randomly selecting two samples and assigning weights $\lambda$ and $1-\lambda$ to each instance, where $\lambda$ is randomly drawn from a beta distribution^38^. The beta distribution is a continuous probability distribution defined on the interval [0, 1]. It is commonly used to model random variables representing proportions or probabilities^43^. The resulting input-outputs are combined using linear interpolation, generating weakly labeled data. Training on weakly labeled data introduces a regularization effect on the ML models, which helps prevent overfitting and improves the neural network’s ability to generalize to unseen data^44,45^. By reducing the reliance on exact (or true/strong) labels and allowing the model to learn from a mixture of samples, Mixup effectively mitigates the impact of class imbalance, where the minority class (in this case, pores) is underrepresented compared to the majority class (non-pores). As a result, Mixup data augmentation strikes a balance between fitting the minority class and the majority class. This avoids underfitting, leading to improved ML model performance. By creating augmented (or weakly labeled) samples that blend the characteristics of different instances, Mixup enables the ML model to capture a broader range of variations. Hence, trained ML models become more robust in detecting potential discrepancies in the collected experimental data. This technique has proven to be effective in various domains, including image classification and segmentation tasks, where the class imbalance is a common challenge^32,38,41,42^. The data augmented sample $(\hat{x}, \hat{y})$ can be represented by:

1 $$\begin{aligned} \hat{x}&= \lambda \times x_{i} + (1-\lambda ) \times x_{j}\nonumber \\ \hat{y}&= \lambda \times y_{i} + (1-\lambda ) \times y_{j} \end{aligned}$$

where $(x_i, y_i)$ and $(x_j, y_j)$, $x_i, x_j \in X$, $y_i, y_j \in Y$, which are strongly labeled samples. *X* is the input time-series vector, and *Y* is the target pore or non-pore labels. As we are working with a classification problem, we assign a value of 1 to $y_i \in Y$ for a pore and 2 for a non-pore. $\hat{y}$ is rounded to the nearest integer during interpolation. This process adds noise to the datasets, forcing the neural network to learn from them.

**Figure 4:**
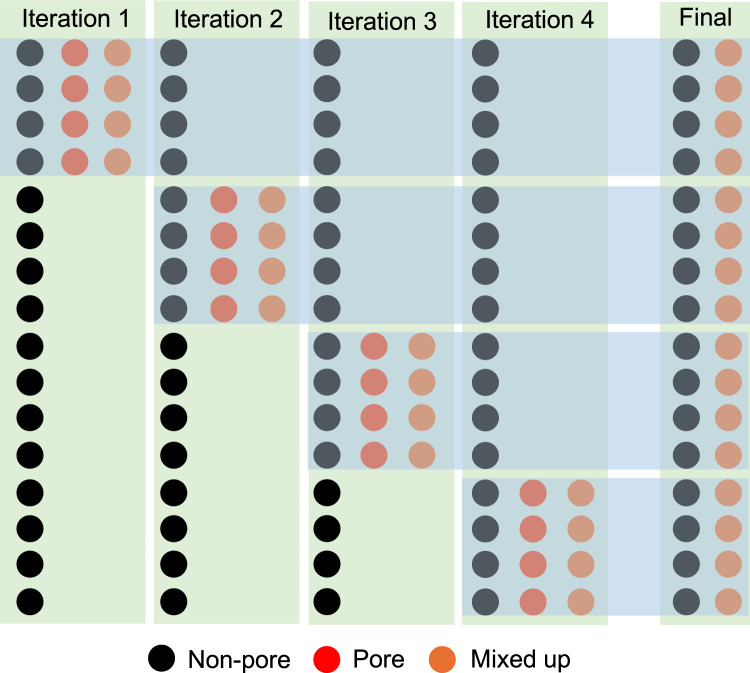
This is a schematic of a non-pore:pore = 4:1 Mixup method, where an equal number of non-pore and pore samples are mixed to generate mixed-up data. This process is repeated until the pore data is mixed with all non-pore data, making the dimensions of non-pore and mixed-up data the same.

**Figure 5:**
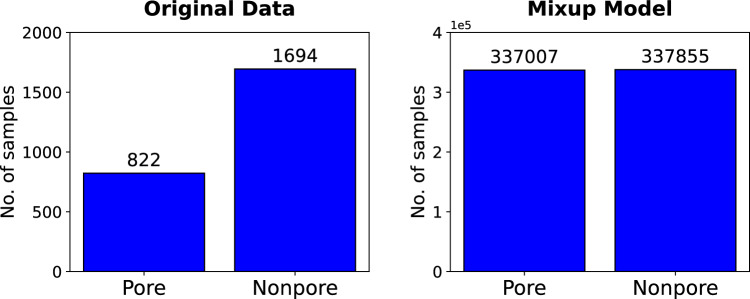
Distribution of pore and non-pore samples before and after data augmentation.

We used the non-pore-to-pore ratios to optimize the number of combinations or ‘mixups’ between two samples with our class imbalance data. By re-using the uncommon event (in this case, pores) multiple times, we effectively increase the size of the training data set. This Mixup-enabled data augmentation strategy accurately trains ML models by reducing the emphasis on a handful of identical rare events, reducing reliance on uncommon events, increasing robustness when learning from corrupt pore/non-pore labels, and improving generalization when faced with adversarial examples. For example, if there are 16 non-pore and four pore samples, the non-pore-to-pore ratio would be 4:1. As indicated in Fig. 4, four iterations of this are performed for each test. In the first iteration, augmented training data is constructed from combinations between the four pore acoustic events and the first four non-pore data. In iteration 2 (Fig. 4), the same four-pore acoustic events are mixed with the subsequent four non-pore events. This Mixup process continues for the next two iterations until an approximate 1:1 ratio number is met. For the combined dataset (Table 2), the original non-pore-to-pore ratio is approximately 2:1. Accordingly, we perform two iterations for this case, which leads to a balance in the data. Figure 5 shows the pore and non-pore sample distribution for the combined dataset before and after this Mixup process. The ratio improves from 2:1 (1694 non-pore and 822 pore samples) to 1:1 (337855 non-pore and pore 337007 samples).

The data augmentation method described in the preceding paragraphs discusses a one-to-one ratio between the unique combination and the synthetically constructed sample (one augmented selection for each combination). Multiple $\lambda$ values were used to generate more samples per combination to increase the sample size further. For example, two different $\lambda$ values will construct two augmented samples for one combination. This research analyzed 1–10 $\lambda$ values for each acoustic set and 1–3 $\lambda$ values for the combined dataset.

Reusing pores many times increases the size of the training data set, facilitating better training of ML models by placing less emphasis on a handful of identical pores. Therefore, a trained ML model barely memorizes the identical uncommon events, which increases the robustness of neural networks when learning from mixed pore/non-pore labels. Moreover, such ML models generalize better when faced with adversarial examples.

### ML-model using CNN

We develop a CNN to learn a mapping function between acoustic raw time series and pore/non-pore labels using the original and augmented training data. The convolutional neural layers and associated kernels allow us to learn this mapping by extracting representative features from the acoustic raw time series. Max pooling was employed to condense the number of abstract features, which are finally connected to a dense layer. It calculates the maximum value in each patch of each feature map. The convolutional layer outcomes are downsampled, and the pooled feature maps provide the most present feature in the patch. Dropout is used in these dense layers to reduce overfitting. Moreover, early stopping was used to overcome overfitting. Mathematically, this CNN architecture for acoustic measurement classification can be described as:

2 $$\begin{aligned} \textrm{output}\left( N_i, C_{\textrm{output}_j}\right) =F\left( \sum _{k=1}^{C_{\textrm{input}}}\varvec{W} \left( C_{\textrm{output}_j},k\right) *\textrm{input}\left( N_i, k\right) +\varvec{b}\left( C_{\textrm{output}_j}\right) \right) \end{aligned}$$

where *N* is the batch size, $C_{\textrm{input}}$ is the number of acoustic measurements in the 10 ms window, $C_{\textrm{output}}$ is the value of pore and non-pore labels (i.e., 2), $\varvec{b}$ is the bias, $\varvec{W}$ is the weight, *F* is an activation function, and $*$ is a valid cross-correlation operator. Specifically, within the context of our problem:

- $\textrm{Output}\left( N_i, C_{\textrm{output}_j}\right)$: Represents the output value at the i-th sample in the batch ($N_i$) for the j-th output channel ($C_{\textrm{output}_j}$). This indicates the network produces multiple outputs corresponding to different pore/non-pore classifications.
- *F*: Activation function that introduces non-linearity. Common choices include ReLU or sigmoid.
- $\sum _{k=1}^{C_{\textrm{input}}}$: Summation over all input channels (k) from 1 to $C_{\textrm{input}}$.
- $\varvec{W}\left( C_{\textrm{output}_j},k\right)$: Weight matrix specific to the j-th output and k-th input channels. This captures how the network learns to combine features from different input acoustic emission measurements.
- $*$: This cross-correlation operator differs from the typical convolution used in CNNs for images. It performs a ‘sliding dot product’ between the filter (weights) and the input, resulting in the same output size as the input. Here, it extracts correlations within the 10 ms window of acoustic emission measurements.
- $\textrm{input}\left( N_i, k\right)$: Represents the k-th acoustic measurement for the i-th sample in the batch.
- $\varvec{b}\left( C_{\textrm{output}_j}\right)$: Bias term for the j-th output channel.

The above Eq. (2) calculates a single neuron’s activation in the CNN’s output layer. The network essentially learns weights ($\varvec{W}$) to combine different acoustic measurements (captured by the input channels) within a 10 ms window using valid cross-correlation. The activation function (*F*) then introduces non-linearity to create a more expressive pore/non-pore classification model.

We minimize sparse categorical cross-entropy loss function ($\mathcal {L}$) to find the best model:

3 $$\begin{aligned} \mathcal {L}(\varvec{y}, \varvec{\hat{y}}) =-\sum _{i=1}^{N}y_{i}\textrm{log}\left({ \hat{y}}_{i}\right) , y_i \in \varvec{y}, \hat{y}_{i} \in \varvec{\hat{y}} \end{aligned}$$

where $\varvec{y}$ is the ground truth, and $\varvec{\hat{y}}$ is the prediction. $y_i$ is the true label (pore or non-pore) for the i-th sample. $\hat{y}_{i}$ is the predicted probability for the i-th sample belonging to the pore class (other classes will have their own probabilities summing to 1).

The Eq. (3) defines the sparse categorical cross-entropy loss function, commonly used for multi-class classification problems. It penalizes the network for making incorrect predictions, aiming to minimize the overall loss during training. Here, the network strives to minimize the difference between the predicted pore class probabilities ($\hat{y}_{i}$) and the true labels ($y_i$).

For the activation function, we used a rectified linear unit (ReLU). ReLU is described as:

4 $$\begin{aligned} f(x) = \max (0, x) \end{aligned}$$

where *x* is an input to a neuron.

**Figure 6:**
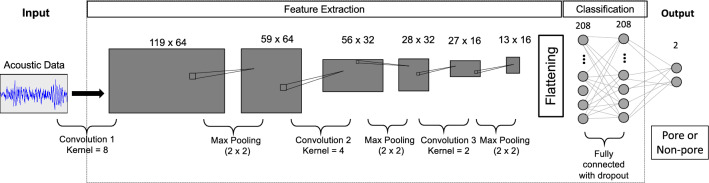
Schematic overview of the CNN architecture employed in this study. We are training a CNN on acoustic emission data to identify whether a signal is a pore or non-pore. The acoustic emission signal is co-registered to the location of individual pores/non-pores.

Figure 6 depicts a schematic of the CNN used in this study, developed after studying similar architectures in literature^46–48^. The input is an acoustic raw time series of size 1000 $\times$ 1. Three convolutional and max-pooling layers perform feature extraction. The three convolutional layers use ReLU activation function with 64, 32, and 16 filter sizes, respectively. After the training samples pass through all these layers, the extracted features are flattened to a 1-dimensional array. This 1D array is then connected to a dense layer with dropout where pore labels are predicted. The output of the fully connected layer is the pore or non-pore classification. Several ML frameworks, such as deep neural networks, convolutional neural networks, and recurrent neural networks, may be employed for pore predictions. These frameworks are all suitable for handling complex datasets. CNN was deemed optimal because its kernels allow us to extract better features than a fully connected dense neural network.

### Evaluation metrics

Accuracy is a metric commonly used for evaluating classification tasks. However, caution must be exercised for class imbalanced data such as those presented in Table 2 since the accuracy metric may be artificially high due to the majority. To have a complete evaluation of the model, precision, recall, and F1 scores are implemented to measure CNN’s performance^49^:

5 $$\begin{aligned} \textrm{Precision}= & {} \frac{\textrm{TP}}{\textrm{TP} + \textrm{FP}} \end{aligned}$$

6 $$\begin{aligned} \textrm{Recall}= & {} \frac{\textrm{TP}}{\textrm{TP} + \textrm{FN}} \end{aligned}$$

7 $$\begin{aligned} \mathrm{F1\ Score}= & {} \frac{\mathrm{(2 \times Precision \times Recall)}}{\textrm{Precision} +\textrm{Recall}} \end{aligned}$$

where TP, FP, and FN refer to true positive, false positive, and false negative. F1 Score is a precision and recall function commonly used to evaluate individual classes in an unbalanced dataset. In this study, a TP is an acoustic event correctly identified as a pore; FP is an acoustic event incorrectly identified as a pore; and FN is an acoustic event that is a pore but incorrectly identified as a non-pore.

## Results and discussion

The ML model built using CNN was applied to the original and augmented acoustic datasets with one $\lambda$ value for data augmentation. We discuss the resulting performances of both classification experiments, summarized in Table 3.

### Impact of data augmentation

**Figure 7:**
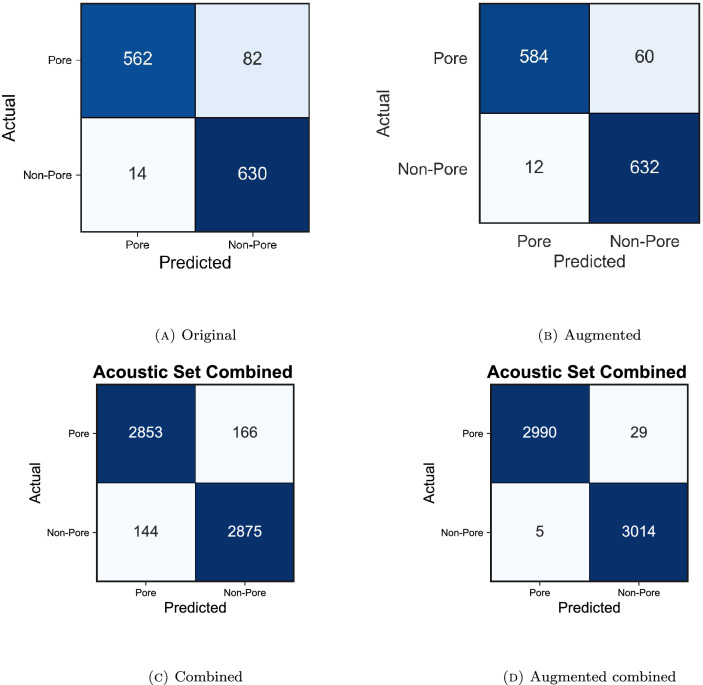
Confusion matrices comparing (**A**) the original dataset and (**B**) the augmented dataset (with one $\lambda$ value) for acoustic set 1. Confusion matrices for the combined (**C**) and augmented combined-acoustic dataset (**D**). The correctly classified samples are along the diagonal, and the misclassified samples are off-diagonal.

Augmentation affects CNN model performance as the evaluation scores slightly increased compared to the original data, with F1 scores and accuracy rising by at least 0.01 across all sets. Moreover, the individual datasets 1, 2, 3, and 4 all slightly increased evaluation scores, although set 5 shows a slight decrease in scores. The combined data returned the highest F1 and accuracy scores for both augmented and original data (Table 3). Note that while these accuracy increases are numerically small (< 0.05), the error rates are substantially improved as the models trained on the original data perform near 0.95 for accuracy and F1 scores as is. With augmented data, all three scores increase to 0.99.

Figure 7 shows the confusion matrix for acoustic dataset 1, which summarizes the classification accuracy. While the F1 score and accuracy for acoustic set 1 increased nominally by a value of 0.01, Figure 7 shows a fairly significant decrease in misclassifications with data augmentation, improving CNN performance on test datasets. In particular, when data augmentation is applied, 24 more samples are classified correctly compared to their original counterpart data. Specifically, 22 more pores and two more non-pores, which were misclassified before data augmentation, are now correctly classified when Mixup is applied. Table  4 details the model performance values for each acoustic dataset before and after data augmentation with one $\lambda$ value. For all but acoustic set 5, TP and TN increased with data augmentation. The combined dataset has the overall best model when data augmentation is applied with a total of 34 misclassifications (Fig. 7).

**Figure 8:**
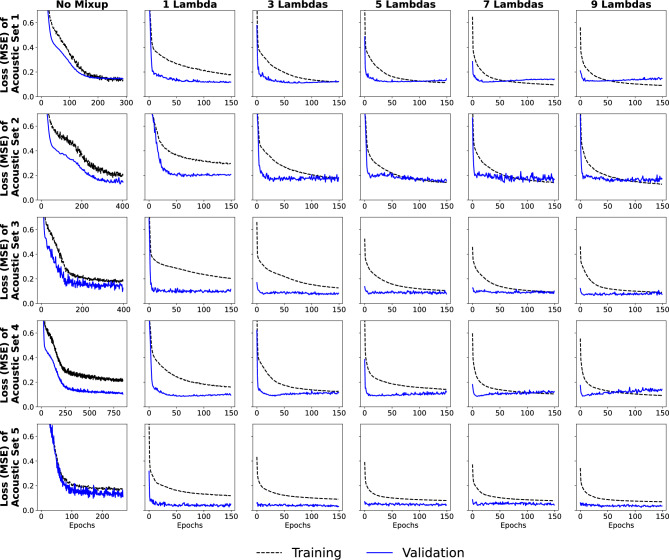
Training and validation loss plots of the five acoustic datasets. Both unmixed and mixed scenarios with up to 9 $\lambda$ values are shown.

Figure 8 depicts the training and validation loss of the five datasets with and without Mixup. For the cases with the Mixup, training details of up to nine $\lambda$ values are shown. Note that the number of epochs used to train the CNN is generally less when applying data augmentation than the original data. For the original data, the number of epochs needed to train the model varies across the acoustic sets, ranging from 150 to over 400, with set 5 requiring the fewest epochs and set 2 requiring the most. In contrast, all acoustic datasets are trained well within 50 epochs with data augmentation. Although training requires fewer epochs for the augmented data, the training time required is more than one magnitude higher for $1 \, \lambda$ (Table 5), which is expected with the increased data in data size. Moreover, the training time for augmented data grows higher for multiple $\lambda$.

**Table 5:** Training time for original and augmented data.

Data set	Training time for original data (s)	Training time for augmented data (s)
Set 1	40.99	829.46
Set 2	28.16	372.96
Set 3	46.62	2884.87
Set 4	33.65	1042.82
Set 5	42.01	5982.22

The time taken to process each acoustic series is in the order of milliseconds. The ML models are trained on a laptop with the following configuration: Intel(R) Xeon(R) CPU E5-2695 v4 2.10GHz with 64GB of RAM and 64-bit processor. Set 1 to Set 5 refer to each acoustic experiment data.

### Influence of $\lambda$ in data augmentation

We now examine the effect of using multiple $\lambda$ values on the misclassification rate. Here, we quantify the classification rate simply by summing TP and FP predictions. Table  6 details the number of misclassifications for each acoustic set with the number of $\lambda$ values. The number of misclassifications is smallest when using only 1 $\lambda$ for all acoustic datasets. Furthermore, with two and higher $\lambda$, there is no consistent trend in the misclassification, but they are all consistently higher than what was found for 1 $\lambda$. Using more than one $\lambda$ value does not necessarily lead to improved modes since it does not decrease the number of misclassified predictions. On the contrary, it increases the number of misclassifications, suggesting a degradation in the CNN performance due to data augmentation. In addition, from Fig. 8, we observe that there is little change in the loss plots after 1 $\lambda$, as all loss plots look similar and reach a minimum loss within 50 epochs. While a single Mixup step improves performance, applying multiple augmentation steps leads to overfitting because subsequent augmentations do not provide sufficiently informative and independent new observations. This redundant information leads to overfitting and reduced CNN’s performance. Such multi-augmentation eventually increases noise in the data. Therefore, trained CNNs fail to predict original test datasets more accurately than a single Mixup step.

**Table 6:** Total number of misclassifications for each acoustic set as more lambda values augment dataset size. More data augmentation iterations provide more misclassifications.

Set	Original	1 $\lambda$	2 $\lambda$	3 $\lambda$	4 $\lambda$	5 $\lambda$	6 $\lambda$	7 $\lambda$	8 $\lambda$	9 $\lambda$	10 $\lambda$
1	96	72	90	75	78	185	112	126	160	122	120
2	101	77	126	95	107	123	131	149	95	150	151
3	65	55	92	75	111	87	78	115	102	115	101
4	125	112	125	167	151	173	143	150	163	152	145
5	57	70	113	119	136	108	121	130	111	113	142

### Dataset size and non-pore to pore ratio

The non-pore-to-pore ratio in the training dataset significantly controls CNN model performance. Acoustic sets 1–4 have non-pore-to-pore ratios between 1.8 and 4.3, whereas acoustic set 5 has a ratio of 1.2. Also, acoustic sets 1–4 show increased model performance with augmented data, as indicated by increased F1 scores and accuracy (Table 3). Acoustic set 5 slightly decreases model performance with augmentation, as indicated by the reduction of F1 scores and accuracy in Table 3. The combined data has the largest training dataset of 2516 independent and informative observations (Table 2). Out of all the acoustic sets, the combined dataset provided the best ML performance with both the original and augmented data (Table 3). The combined dataset also significantly increased evaluation scores compared to the original data after the implementation of data augmentation. This is because the combined set has more independent and informative pore and non-pore events than individual sets.

## Conclusions

This study used a CNN-based ML model to predict the formation of pore/non-pores in the AM processes for acoustic datasets that were spatially registered ex situ to pore locations. CNNs were developed for original datasets and on augmented (up to 10 times) datasets to examine the efficacy of data augmentation for the intrinsically sparse datasets available to study pore formation. For each dataset, the performance of a single augmentation was better than multi-augmentations (e.g., 2–10), indicating that an optimal augmentation is achieved with $1 \lambda$ for the considered data. However, multi-augmentation increased dataset size and training time without necessarily increasing accuracy.

Our results indicate that data augmentation had advantages and disadvantages (when the dataset is nearly balanced) on ML model performance depending on non-pore-to-pore ratio and dataset size. Generally, the ML models for augmented datasets perform better than corresponding original datasets except for acoustic dataset 5. For dataset 5, the Mixup strategy did not improve results as the dataset is close to balance (non-pore to pore ratio of 1.2). This may be a limitation of the Mixup strategy for already balanced datasets. The CNN trained on the combined dataset performed best, with an overall performance of 95% and 99% for original and augmented datasets, respectively. Even though the non-pore-to-pore ratio of the combined set is similar to acoustic set 3, this CNN model performed the best after data augmentation, potentially because of the larger sample size and more informative observations compared to individual datasets.

In AM processes monitoring, registered datasets such as the one considered herein are expensive to collect, highly imbalanced, and notoriously sparse in observation counts. Hence, this augmentation methodology can greatly enrich the information extracted from the experimental recordings. Furthermore, this enhanced data has been shown to effectively produce models with improved performance, indicating that practical implementations of such models could be achieved without the traditional burden of large amounts of training data required for neural network approaches. However, while we have shown when to use augmentation or not, the question remains on how we can utilize such CNNs during real-time AM processes. Once a CNN model is trained, a test or unseen acoustic emission signal is inferred in milliseconds. This makes trained CNNs attractive for deployment on a smart computing device (e.g., Raspberry Pi) attached to the data acquisition systems to monitor changes in pore formation during the LBPF process. We may need to retrain a CNN model (or fine-tune it with minimal data) for a different AM system. Future work could investigate the trained ML model on a system with a different setting, transfer knowledge to the existing system for our CNN model configuration, and investigate the potential benefits of hyperparameter tuning in CNN models.

## Data Availability

The datasets and codes used and/or analyzed during the current study are available from the corresponding author (Maruti K. Mudunuru; Email: maruti@pnnl.gov) upon reasonable request.

## Abbreviations

AM: Additive manufacturing
CNN: Convolutional neural networks
CPU: Central processing unit
FP: False positive
FN: False negative
LPBF: Laser powder bed fusion
ML: Machine learning
NMF*k*: Non-negative matrix factorization with custom *k*-means clustering
PSD: Power spectral density
RAM: Random access memory
SVM: Support vector machines
TN: True negative
TP: True positive
